# Aligning systems science and community-based participatory research: A case example of the Community Health Advocacy and Research Alliance (CHARA)

**DOI:** 10.1017/cts.2018.334

**Published:** 2019-02-05

**Authors:** Melinda M. Davis, Paul Lindberg, Suzanne Cross, Susan Lowe, Rose Gunn, Kristen Dillon

**Affiliations:** 1 Oregon Rural Practice-based Research Network, Oregon Health & Science University, Portland, OR, USA; 2 Department of Family Medicine, Oregon Health & Science University, Portland, OR, USA; 3 OHSU-PSU School of Public Health, Oregon Health & Science University, Portland, OR, USA; 4 Community Health Advocacy & Research Alliance, Hood River, OR, USA; 5 Hat Creek Consulting, Providence Hood River Hospital, United Way-Columbia Gorge, Hood River, OR, USA; 6 Community Advisory Council, PacificSource Columbia Gorge Coordinated Care Organization, The Dalles, OR, USA; 7 Columbia Gorge Health Council, Hood River, OR, USA; 8 Clinical Advisory Panel, PacificSource Columbia Gorge Coordinated Care Organization, The Dalles, OR, USA; 9 PacificSource Columbia Gorge Coordinated Care Organization, Hood River, OR, USA

**Keywords:** Community-engaged research, learning healthcare systems, systems science, community-based participatory research, network resiliency

## Abstract

Partnered research may help bridge the gap between research and practice. Community-based participatory research (CBPR) supports collaboration between scientific researchers and community members that is designed to improve capacity, enhance trust, and address health disparities. Systems science aims to understand the complex ways human-ecological coupled systems interact and apply knowledge to management practices. Although CBPR and systems science display complementary principles, only a few articles describe synergies between these 2 approaches. In this article, we explore opportunities to utilize concepts from systems science to understand the development, evolution, and sustainability of 1 CBPR partnership: The Community Health Advocacy and Research Alliance (CHARA). Systems science tools may help CHARA and other CBPR partnerships sustain their core identities while co-evolving in conjunction with individual members, community priorities, and a changing healthcare landscape. Our goal is to highlight CHARA as a case for applying the complementary approaches of CBPR and systems science to (1) improve academic/community partnership functioning and sustainability, (2) ensure that research addresses the priorities and needs of end users, and (3) support more timely application of scientific discoveries into routine practice.

## Background

Community-based participatory research (CBPR) and systems science offer complementary approaches that are designed to engage stakeholders in co-learning and to facilitate the application of research into routine practice [1–3]. CBPR is an approach to research based on equitable involvement of academic and community partners that is designed to improve capacity, enhance trust, and address health disparities [1,4]. Systems science aims to understand the complex ways in which human-ecological coupled systems interact and to apply knowledge to management practices [5]. BeLue *et al.* suggest that CBPR can “benefit from using system[s] science framework to (a) *visualize and specify* the complex and dynamic characteristics of problems faced by community residents and (b) *identify* intervention points and potential “tipping points,” or points at which a community can change from one phase (disproportionate burden of disease) to another (lower burden of disease)” [6]. Similarly, Raymaker (2016) and Silka (2010) suggest that systems scientists could benefit from using CBPR principles and considerations for operationalizing equitable stakeholder engagement in the co-creation of research [1,2].

However, as summarized in Table 1, only a small number of articles discuss CBPR and systems science concurrently [1–3,6]. We posit that systems science and CBPR are synergistic. Systems science provides CBPR researchers tools to inform and guide how partnerships change over time, and a set of theories and tools that can help build resilience. CBPR provides systems scientists with the approach needed to build robust, trusting, and mutually beneficial research partnerships. This article describes the orienting principles of CBPR, identifies key theories and tools used in systems science, and explores the interface of CBPR and systems science to inform a case study of 1 CBPR partnership: the Community Health Advocacy and Research Alliance (CHARA). Linking CBPR and systems science provides tools for partnerships to keep their core identities intact while responding to opportunities and co-evolving with individual members, community priorities, and a changing healthcare landscape. Application of these complementary approaches can (1) improve academic/community partnership functioning and sustainability, (2) ensure that research addresses the priorities and needs of end users (e.g., patients, clinicians, community), and (3) support more timely application of scientific discoveries into routine clinical and community-based practice.

**Table 1:** A brief review of articles linking CBPR and systems science

Author	Summary
BeLue *et al.* (2012) [6]	The authors posit that systems science tools can help CBPR stakeholders to (a) visualize and specify the complex and dynamic characteristics of problems faced by community residents and (b) identify intervention targets or “tipping points” by which communities can transform their health conditions. The authors describe the use of causal loop diagrams by the Clinton County Healthy Communities Community Health Outreach Coalition (CCHC), a community coalition engaged in CBPR activities regarding youth drinking reduction and prevention. The CCHC is a partnership between local county-level agencies and the Clinton County Cooperative Extension and is affiliated with the 2006 Pennsylvania Department of Health State Improvement Plan. The CCHC coordinates community agencies to address issues related to substance abuse, teen pregnancy, and the social determinants of health. The CCHC meets monthly and consists of a volunteer board and subcommittees. The authors suggest that causal loop diagrams can provide an initial basis for simulation modeling in which multiple futures can be explored and leverage points can be identified. They further argue that behavior over time graphs and concept mapping may be helpful tools for CBPR partnerships. They note that training CBPR stakeholders in the proper use of systems science tools is needed.
Silka (2010) [2]	The author argues for the alignment between scholars that are using CBPR in health with the work of systems (sustainability) scientists. Both orientations are focused on addressing challenges that result from “nonparticipatory research.” She suggests that CBPR and systems science are focused on the coproduction of knowledge that offers solutions to complex problems, and both approaches may be criticized by “traditional” scientists because of their problem focus and lack of a positivist approach. The author suggests that concepts and tools from systems science such as wicked problems or agent-based modeling may be helpful in CBPR efforts. The author highlights the activities of the University of Main Sustainability Solutions Initiative (SSI), which “brings together multiple disciplines and many stakeholders to work on a range of coupled problems that must be solved if Mainers’ way of life is to be sustained economically, environmentally, and culturally” in order to identify challenges in stakeholder-engaged research in systems (sustainability) science, which can then help CBPR researchers see their challenges in new ways. The examples illustrate the use of structured dialogues, futures, and agent-based modeling, and using GIS to inform scale up of strategies and solutions. She introduces the concept of “wicked problems” in SSI discussions, which she defines as complex problems “in which stakeholders are likely to hold conflicting interpretations of what the real problem is and what the causes are, and they bring to the problem different values, goals, and life experiences.” In contrast to “tame problems” which can be addressed by gathering data, in addressing wicked problems it may never be clear when sufficient data has been amassed to point to a workable solution. Silka uses various examples to highlight how CBPR may need to shift from a focus on addressing inequalities in power to one focused on a problem-centered approach; she argues that practitioners will need new tools to deal with the complexity of community problems even when power issues have been addressed. Additionally, Silka argues that incentive structures need to change in order to support actual impact of research on practice, rather than just publication in “high-impact” journals.
Frerichs *et al.* (2016) [3]	The authors posit that CBPR and systems science can help address lingering questions about racial and socioeconomic health disparities. They summarize the history and principles of systems science and CBPR and highlight five synergistic properties of integrating these approaches: (1) paradigmatic, (2) socioecological, (3) capacity building, (4) co-learning, and (5) translational. Specifically, they describe how qualitative techniques (e.g., building “mental models” through causal loop diagrams or creating stock and flow diagrams) and computational techniques from systems science (e.g., using agent-based modeling or network analysis) may be useful to CBPR.
Raymaker (2016) [1]	This manuscript describes what one approach to systems thinking, critical systems thinking (CST), might be able to use from CBPR to inform emancipatory practices and methods for generating more equitable practices. The author provides a brief review of CST and CBPR approaches and highlights the complementary aspects of these orientations, noting that they are “general approaches to social inquiry, methods-agnostic, and use systematic (in CBPR, ‘ecological’) lenses in their approach to identifying, understanding, and intervening in social problems” and they share a focus on emancipatory practices, holism, underlying pragmatism, and focus on “wicked problems” or “mess” areas. Raymaker describes a hybrid approach used by her team to align CST and CBPR principles in the development and operation of Academic Autism Spectrum Partnership in Research and Education (AASPIRE). AASPIRE first formed in 2006 with a primary purpose to “conduct academic research studies in collaboration with the Autistic community and its allies.” Raymaker describes organizational and management practices used to support engagement and decision making as informed by CBPR and Senge’s concept of the learning organization and the “five disciplines.” Raymaker identifies four ways in which CBPR principles were operationalized: communication processes, material translation, shared decision-making processes that account for power, and using a cyclical and iterative process. She illustrates the use of Senge’s five disciplines, including: shared vision, team learning, mental models, personal mastery, and systems thinking.

CBPR, community-based participatory research.

## CBPR to Bridge the Research to Practice (and Practice to Research) Gap

The gap between known evidence-based interventions and the care delivered in practice is one of the most pressing challenges in healthcare. Nearly 2 decades ago, the Institute of Medicine (IOM, now National Academy of Medicine) identified 2 gaps in research translation: (1) moving basic science discoveries into clinical research (bench to bedside) and (2) moving from clinical research into medical practice and health decision-making (bedside into practice) [7,8]. In response to this challenge, approaches supporting partnered research have blossomed, including practice-based research networks [9,10], the Clinical and Translational Science Award (CTSA) program [11,12], and various strategies for community/stakeholder engagement in research [13,14].

CBPR, which builds on the work of Paulo Freire [15], is notable as an approach to partnered research through its core principles of stakeholder engagement, co-learning, and balance between research and action. CBPR supports equitable involvement of scientific researchers and community members in the research process, acknowledgement of partner strengths, ongoing commitment, and an iterative approach to knowledge creation and system change over time. The 9 principles of CBPR first identified by Israel and colleagues are summarized in Appendix 1 [1,4]. While principles capture the core values of CBPR, application may vary related to how a community is identified, details about the collaborative partnership(s), and level of responsibility for project activities [4].

Authentic partnerships between the community and the researcher and balance between research and action toward ending health disparities are often considered standards for assessing CBPR approaches [4,16,17]. Many articles describe how CBPR partnerships have been developed with vulnerable communities (e.g., rural, racial/ethnic populations, low income), and the methods frequently involve leveraging existing community infrastructure, assessing and identifying priority areas, and implementing a target project/intervention [13,18,19]. A number of CBPR initiatives focus on a single disease (e.g., diabetes, asthma, obesity) rather than a broad spectrum of factors impacting community health. Although long-term relationships and commitment are principles of CBPR, few articles highlight how to sustain CBPR partnerships or describe evolution over time [20–22]. This is driven in part because research funding rarely includes financial support necessary to sustain CBPR partnerships beyond individual studies. Despite academic partner intentions, this funding structure reinforces helicopter research—the process by which academicians fly into communities, take needed data, and give nothing back—and the erosion of community trust [23].

## Systems Science to Support Problem Identification, Tipping Points, and Organizational Resilience

Systems science provides ideas and tools that can help CBPR partnerships identify intervention priorities, inform and guide how partnerships change over time, and make strategic investments to build resilience. Systems science is an interdisciplinary field of study that is conceptually grounded in exploring the component parts of a system, their interrelationship, and their relationship to a functioning whole, a design perspective commonly referred to as “systems thinking” [3,6]. Systems science has largely developed as a field of inquiry and practice in the 20th century with roots in disciplines such as biology, anthropology, physics, psychology, mathematics, management, and computer science [5]. Informed by the thinking of founders of the systems movement like Ludwig von Bertalanffy [24], complex systems and complexity science are fields within systems science. In practice, systems science is a nonreductive approach that is intended to improve the quality of a system as a whole, its parts, and the interactions within and between levels of the system (i.e., focal scales) [5]. Systems scientists explore the impact of feedback, unintended and long-term effects, chaotic dynamics, and emergent behaviors on systems using various theories, methods, and tools [1,5]. Approaches used in systems science include critical systems thinking and sustainability science among others [1,2].

Systems science is increasingly being applied to healthcare and public health [5,25]. Both “hard” systems methodologies (e.g., quantitative dynamic model building) as well as “soft” systems methodologies (e.g., qualitative, action-based methods) may be useful for understanding the interaction between system components, the relationships between them, and emergent complex behaviors [25]. In his 2014 article, Peters reviewed a number of systems thinking theories, methods, and tools that can be useful in healthcare—and CBPR—such as learning organizations theory, punctuated equilibrium, agent-based modeling, and causal loop diagrams. He noted:

*The theories and methods in systems thinking are each designed to address complex problems. They are complex because they involved multiple interacting agents, the context in which they operate keeps changing, because the manner in which things change do not conform to linear or simple patterns, or because elements within the system are able to learn new things, sometimes creating new patterns as they interact over time* [5].

Similar to CBPR practitioners, systems scientists use research to study problems in ways that lead to application, action, and solutions [2]. However, early approaches to systems thinking did not always attend to the influence of power. For example, Raymaker wrote that, “systems thinking and its methods can be exploited by those in dominant positions—either deliberately or through lack of awareness—to maintain the status quo” [1]. Others note that systems scientists can be challenged by the gap between research discovery and application in practice. For example, Silka profiled work by Cash *et al.* (2006), which likened the production of scientific knowledge to a loading dock—with the assumption that once the research occurred, there would be an immediate market for the product [2].

## CHARA Case Example: Establishing and Sustaining a Rural CBPR Partnership

In 2013, with seed funding from the Patient Centered Outcomes Research Institute (PCORI) Pipeline to Proposal Program, Kristen Dillon, MD, and Melinda M. Davis, PhD, initiated an academic/community research partnership that became the CHARA. This partnership was informed by the CBPR approach and focused on using research to complement and leverage health system transformation to enhance health and address disparities in the Columbia Gorge region (see Appendix 2). The Gorge region spans the Columbia River and includes seven counties located in Oregon and Washington. The Gorge is home to 85,000 residents dispersed over 10,000 square miles of rural, frontier, and tribal land. Only two cities in the region (i.e., Hood River and The Dalles) have populations of more than 5000 residents. We summarize CHARA development and concurrent health system transformation here; additional details on concurrent regional activities are available in Dillon *et al.* (in press) [26].

**Fig. 1:**
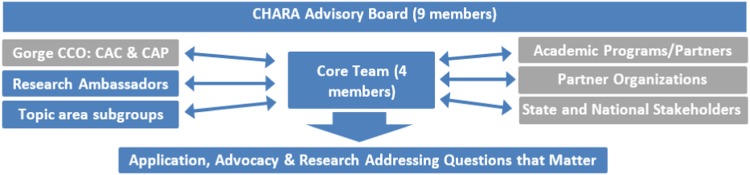
Community Health Advocacy and Research Alliance (CHARA) Organizational Structure and Key Collaborators. Abbreviations: CCO=Coordinated Care Organization, CAC=Community Advisory Council, CAP=Clinical Advisory Panel.

Over time, Drs Dillon and Davis developed the CHARA network infrastructure as described in Fig. 1, which includes leadership from the core team (i.e., Dillon, Davis, Lowe, and Network Manager) and oversight by a 9-member advisory board. Dr Dillon was a practicing primary care clinician and long-term resident of the Gorge who had helped initiate formation of the region’s Medicaid Accountable Care Organization (see additional detail in Appendix 2). Dr Davis was a faculty member at a nearby university (Oregon Health & Science University, OHSU) who had a history of working with partners in the Gorge—first as a student from the region, second as a practice facilitator for the Oregon Rural Practice-based Research Network (ORPRN), and third as a participatory implementation scientist with a focus on reducing rural health disparities [27,28]. Ms Lowe worked in senior services before retirement and now serves as a patient advocate and stakeholder on numerous initiatives regionally and nationally. Ms Lowe became interested in CHARA after participating with Dr Dillon in a national training on Patient and Clinician Engagement. The network manager has changed over time, based on personal preferences and funding; however, to support local control and power sharing aligned with CBPR, it has been a priority to hire someone from the local community. The CHARA advisory board includes the core team and patient, community, and health/service organization representatives (see “Understanding Priorities” section).

During the first year of CHARA funding, the core team and advisory board worked together to craft our mission, vision, and values statement (see Appendix 3). The core team also built relationships with our growing network of research ambassadors, organizational affiliates, and academic partners. Research ambassadors were individuals (e.g., patients/community members, service providers, health system leaders) who wanted to stay informed about network activities and to participate in studies related to their stated areas of personal/professional interest. Organizational affiliates were existing programs (e.g., schools, public health, service agencies) that expressed interest in collaborating on topic-related grant applications. Academic partners where those with shared areas of interest, who also displayed the ability to work with network partners using collaborative, equitable, service-oriented approach. In the last 4 years we have pursued 3 parallel and complementary lines of work in order to build CHARA: understanding priorities, building capacity, and pursuing research and action. We briefly describe these activities in the following sections.

### Understanding Priorities

Our goal was to leverage rather than replicate existing infrastructure in the Gorge region. Although CHARA originated with a broad focus on improving community health, many funders target specific diseases or conditions. In order to get the lay of the land needed to identify regional priorities, our core team pursued 4 activities. First, starting in 2014, we made presentations to regional advisory boards (e.g., The Next Door, Inc, Head Start, Rotary) in order to describe CHARA’s mission and vision, listen to what these established community organizations were currently doing, and to discuss areas of interest or need. Second, we conducted appreciative inquiry interviews between March and September 2014 with a purposive sample of 27 community stakeholders that represented the age, gender, and ethnic make-up of the region. Appreciative inquiry is an emerging research methodology and organizational development intervention that departs from the problem-oriented research paradigm to study strengths and resources and to recognize successful practices—a way to systematically investigate what gives “life” to a system [29]. Thus our semi-structured interview guide focused on eliciting the factors that created health and were seen as regional assets, as a way to supplement illness and barrier-focused information from a recently completed regional health needs assessment. Three overarching themes emerged from these interviews: (a) health is defined as a multidimensional concept that encompasses characteristics of the individual and local community, (b) preventive behaviors are valued as a way of staying healthy across the lifespan, and (c) personal and community resources provide opportunities for individuals to foster health [30]. Third, we recruited patients, community members, and key health/service partners to serve on our advisory board. Board members were selected based on their interest in academic/community partnered research; representation of key organizational partners, populations, and regional communities; and commitment to healthy, productive collaborative relationships. Finally, representatives from the core team and CHARA board attended monthly meetings of the Community Advisory Council starting in 2015 to stay abreast of regional priorities and changing needs.

Findings from these 4 modes of data collection/outreach were reviewed iteratively by the core team and the board to understand priorities. At the end of the first year of CHARA activities, we identified 3 priority areas for action and research: (1) fostering healthy lifestyles through diet/physical activity or addressing social determinants of health, (2) conducting research or programs to improve delivery of clinical quality measures (e.g., colorectal cancer screening), and (3) improving access to mental health/substance misuse prevention and treatment services. Over time, CHARA has continued to evolve our research priorities to align with the regional Community Health Improvement Plan (CHIP). A community health assessment is conducted every 3 years to inform the CHIP, which forms a solid basis of CHARA’s work, as the CHIP design and implementation involves more than 30 community partners, consumers of Medicaid, and other communities that people in power often overlook. As a community tool, the CHIP is utilized to steer programmatic and research funding, which must be done collaboratively.

### Building Capacity

Concurrent with outreach to build the network and understand regional priorities, our core team engaged in multiple activities to build the capacity of community partners to engage in research. This included hiring local staff to work with CHARA, delivering targeted workshops/trainings, and using projects and research studies as “teachable” opportunities. First, we hired and trained staff locally. This included the CHARA network manager and other staff later engaged in funded projects (see research and action). Second, we developed and delivered a series of community-based research trainings. These trainings leveraged prior materials developed by Dr Davis to support academic/community research partnerships through Community Health Improvement and Research Partnership training materials (see http://communityresearchtoolbox.org/) [13] and leveraged a request to deliver regional Patient and Clinician Engagement symposia (see http://www.napcrg.org/PatientEngagment).^a^ In May 2015, we hosted a 2-hour regional symposium in Hood River, Oregon (one Gorge community) on research methods and advocacy which was attended by ~10 stakeholders. We adapted materials from Community Health Improvement and Research Partnership and Patient and Clinician Engagement to align with local interests and training time. Topics included a review of research, a summary of research methods (including comparative effectiveness research, PCORI’s priority area of funding), and interactive exercises that used the Population, Intervention, Comparator, Outcome, Timing, Setting framework to refine areas of interest into researchable questions. The first session was so well received that a second session was added and attended by ~23 stakeholders in June 2015 in a neighboring Gorge community (i.e., The Dalles, Oregon).

Building on this experience, and at the request of CHARA board members, we held a CHARA Sponsored Community Research Retreat on a Saturday in November 2015. Four objectives were identified for this retreat: (1) strengthen the CHARA partnerships, (2) initiate community/academic relationships and collaborations, (3) refine and develop research ideas for 3-4 identified topic areas, and (4) identify areas for future research project development. The CHARA retreat was held in The Dalles, Oregon and attended by 20 community and academic partners. The core team and CHARA advisory board selected The Dalles to encourage attendance of community-based partners based on travel proximity and accessibility. The agenda was built around developing relationships, sharing CHARA’s history and mission, reflecting on what makes academic/community partnerships work, and engaging in discussions regarding research collaborations focused on the 3 priority areas identified in CHARA’s prior work (see “Understanding Priorities” section). On the retreat evaluation 1 community stakeholder wrote, “it was a very safe and inviting space to learn and ask questions that can lead to collaborative partnership.” Another CHARA 2015 retreat participant reflected:

*I don’t know what was on your list of 30 rules [for collaboration], but I bet that we followed them all. That’s what true collaboration looks like. A friend of mine was in [the area] for a [another] research conference. We shared stories of our day and we both agreed that mine won hands down. It really changed my outlook in ways I didn’t expect. Thank you for including me*.

### Action through Research and Service

CHARA has always focused on aligning research toward action, a core principle of CBPR [4]. Prior work by our team suggested that community stakeholders are motivated by solving problems when answers are known and by designing studies when there is uncertainty [31]. We have done this by utilizing a tiered partnership infrastructure, holding regular meetings to reflect on community needs, identifying known solutions, and developing researchable questions. Specifically, the core team has met weekly or bi-weekly since CHARA inception, and the CHARA board has met monthly or quarterly depending on project demands and funding. CHARA research ambassadors, organizational affiliates, and academic collaborators have been engaged when resources allowed through a network newsletter, attendance at Community Advisory Council meetings, and stakeholder subgroups related to specific interest areas. The network has also focused on blending and braiding funding from research grants, project grants, and contracts. Table 2 summarizes the portfolio of CHARA awards, the topic area of focus, and the lead partner (i.e., community, academia).

**Table 2:** CHARA-related awards, 2014-present*

Title	Topic	Year awarded	Length	Held by	Amount	Funder
PCORI Tier I	Capacity building	2014	<1 year	Community	$15K	PCORI
InteGREAT	Integrated care	2014	1 year	Academic	$250K	Columbia Gorge Health Council
AHRQ PCOR K12	PCOR & CRC	2014	2.5 years	Academic	$305K	AHRQ/OHSU
PCORI Tier II	Capacity building and CER	2015	1 year	Community	$25K	PCORI
Finding the right FIT	CRC	2015	1 year	Community	$25K	Knight CPP
PCORI Tier III	Capacity building, CRC	2016	1 year	Academic	$50K	PCORI
Mobilizing action for resilient communities (MARC) Evaluation	Trauma informed care	2017	2 years	Community	$14K	RWJF
PRECISE CRC	CRC	2017	4 years	Academic	$682K	NCI

AHRQ, Agency for Healthcare Research and Quality; CER, comparative effectiveness research; CHARA, Community Health Advocacy and Research Alliance; CMS, center for Medicaid and Medicare services; CPP, community partnership program; CRC, colorectal cancer; FIT, fecal immunochemical test; NCI, National Cancer Institute; OHSU, Oregon Health & Science University; PCOR, patient-centered outcomes research; PCORI, patient-centered outcomes research institute; RWJF, Robert Wood Johnson Foundation; SDH, social determinants of health.

* In addition to these awards, between 2014 and present, the Community Health Impact Specialist who serves on CHARA’s board has secured over $7 million in community-based projects.

Many of CHARA’s early awards focused on capacity development and on implementing interventions to improve colorectal cancer screening. This was a strategic decision by the CHARA core team and board because (a) colorectal cancer screening was one of the clinical quality metrics identified as a community priority area, (b) the interventions are known but underused in rural and low-income populations, (c) screening is a target for improvement on a national level, and (d) it was seen as a “less wicked problem” that could serve as low-hanging fruit for partner funding and ongoing success. For example, “Finding the Right fecal immunochemical test (FIT)” was a mixed-methods CHARA-led study that explored the voice of the consumer (patient) in the selection of FITs for colorectal cancer. Although multiple studies evaluate the clinical effectiveness of FITs, this was the first study to provide comparative data on patient preferences for FIT characteristics (e.g., collection tool, number of samples, instructions) [32].

CHARA was highly successful in hitting the benchmarks for the PCORI Pipeline to Proposal Program. We developed network governance, identified research priorities, and submitted a full application to PCORI in December 2016 to partner with 5 additional rural regions to compare the effectiveness of 2 strategies to improve fecal testing for colorectal cancer. The application was reviewed but not awarded, and partners are in the process of making revisions to support resubmission. In the interim, CHARA partners have been able to secure other forms of funding, including a National Cancer Institute career development award that Dr Davis leads. Additionally, from 2014 to the present, the Gorge’s Community Health Impact Specialist (Paul Lindberg), who also serves on CHARA’s board, has secured more than $10 million in project-related funding for community stakeholders to address priorities identified in the regional CHIP. He also helped the Gorge region secure recognition in 2016 for the Robert Wood Johnson Foundations’ Culture of Health Prize. Additionally, CHARA activities garnered recognition for Dr Davis as the 2016 Emerging Leader for the Oregon Public Health Association.

## Applying Systems Science Concepts to Manage CHARA Resilience/Sustainability

Although the awards and activities of CHARA are promising, we experienced a gap in funding in mid-2017 with the end of PCORI Tier III award. Core team and board members committed to maintaining basic network functioning, and in Fall 2016 CHARA supported OHSU’s CTSA resubmission, which would fund a part-time community liaison for the Gorge and a portion of Dr Davis’s effort. However, the community liaison will report to the CTSA community engaged research core, not to Drs Davis or Dillon who originated CHARA. The OHSU CTSA award was funded in 2017 and CHARA board members are currently working with CTSA leadership to clarify the role the community liaison will play, such as supporting community-based needs for project evaluation, taking over organizational responsibilities for managing CHARA, engaging research partners around community-identified topics of interest, continuing to elevate the voice of the consumer in research, and supporting diversity in the way research is collected and shared. These factors have amplified the importance of attending to CHARA sustainability and resilience in relation to funding, partner demands, and a changing healthcare and community landscape.

**Fig. 2:**
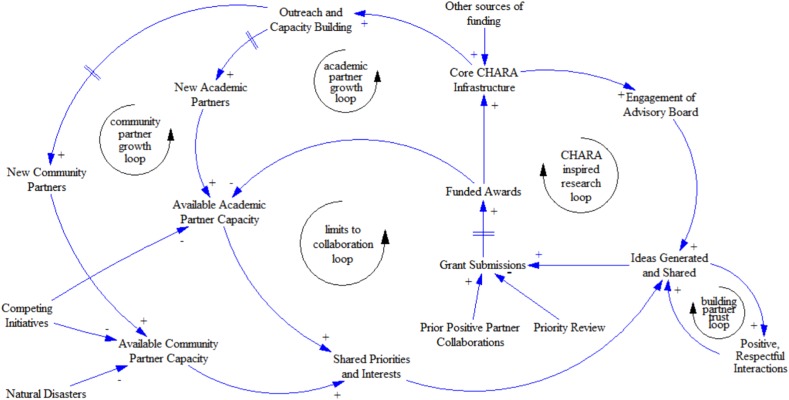
A causal loop diagram of factors contributing to Community Health Advocacy and Research Alliance (CHARA) collaboration and sustainability. Note: In this figure, we use the term “community partner” to refer to diverse stakeholders in the community who are engaged with CHARA – such as individual patients, primary care clinicians, representatives from community-based organizations, and members of cross-agency coalitions.

The tools from systems science shed light on how CHARA—and other CBPR partnerships—could allocate resources to build a robust, resilient structure that allows such networks to maintain their core functioning and purpose while responding to changing environmental and contextual demands. We present a causal loop diagram for CHARA functioning in Fig. 2 and explore how key concepts from systems science could inform partnership activities to support resilience and sustainability.

### Balancing and Reinforcing Loops

Systems scientists frequently articulate the importance of making mental models visible to help identify potential leverage points within a system [33,34]. Tools to help this process can include the construction of stock and flow diagrams or causal loop diagraming, as both aid in visualizing how different variables are connected and interrelated in a system and can subsequently inform simulation models. These diagrams display relevant variables (e.g., nodes or stocks) and the relationship between them (e.g., edges or flows). Relationships can either be positive or negative—indicating how a change in 1 variable affects the related variable. In a closed system, feedback loops may emerge that can be either reinforcing (i.e., positive feedback loop) or balancing (i.e., negative feedback loop) [33]. As depicted in Fig. 2, CHARA primarily operates with reinforcing feedback loops, with limits to growth provided by available academic and community partner capacity. Core CHARA infrastructure, funded awards, and grant submissions are key variables in this model. The “CHARA-inspired research loop” demonstrates how core CHARA infrastructure supports engagement of the advisory board, which leads to ideas generated and shared (which is also influenced by shared academic-community priorities and positive, respectful partner interactions), grant submissions, and ultimately funded awards which reinforce the cycle. This diagram also points out opportunities to enhance resiliency and create “balance” for our system, such as by:

∙ working to identify more controlling loops which kick in if “things go wrong” and advancing this diagram into a hybrid stock and flow model to help assess CHARA capacity, needs, and proactive identify and address potential disruptions;
∙ building redundancy into these partnerships, so they are not as dependent on individuals. This may be accomplished by expanding board membership and engaging additional community and research partners in CHARA activities;
∙ creating a standard budget template that would factor in support for continued infrastructure for the core team (perhaps through indirect cost payments). It is especially important to identify a network manager who can help support the communication processes needed to foster productive academic/community partner exchanges;
∙ leveraging our structure as a self-organizing system to enable diversification and strategic action by area subgroups. The CHARA core team and board are well established. As we identify priority areas we could find ways to designate a community lead, recruit an academic partner, and engage our community-based research ambassadors who have topic interest/expertise but have yet to be tapped. If we continue to embody and practice basic rules by creating space for the partners to interact, to identify priorities, and to support grant and project submissions, we have the potential to secure continued funding.
∙ understanding that the level of contact and engagement with network members may vary over time. This may be shaped by the urgency of the problem or the deadline and the level of social capital (i.e., relationships/trust) that are in place before the target deliverable (i.e., grant deadline). For example, we may be able to pursue some funding opportunities rapidly because the priority has been identified, the focus/intervention selected, and the partners are in place and waiting for an opportunity.
∙ applying concepts encouraged by systems scientist Margaret Wheatley, such as creating process structures, expecting emergence, and evaluating on a multidimensional rather than a linear scale [35]. Although CHARA may transform, the roots and principles are likely to shape the behaviors of academic and community partners across time.

### Adaptive cycle(s)

Walker and Salts articulate the importance of adaptive cycles and focal scales throughout their text [34]. After nearly 4 years of existence, CHARA is in a developmental front loop—moving between rapid growth and conservation. This is an exciting time but also one of potential vulnerability as the network expands partnerships and focus based on initial success. For example, we have built trusting relationships, created network infrastructure, and had success with research and project funding. Some CHARA board members are eager to align local activities with subsequent grant (and evaluation) submissions and to find a way to make CHARA “something,” perhaps by creating a nonprofit or community-based research collaboration. Additionally, a founding partner (Davis) is trying to determine the role for a PhD academic partner when the network is focused broadly on community health, but academic progression encourages specialization. Further, the potential funding for a community liaison through a new collaboration with the CTSA contributes to novel infrastructure and opportunities but may present challenges in aligning goals and demands from another leadership team. Having the core team and board revisit CHARA goals and governance structure presents an opportunity to clarify the network’s core mission and identity which can be used as a touchstone for opportunities moving forward.

### System Archetypes

Archetypes are produced when system structures create common patterns of problematic behavior. Awareness of these problem-generating structures can help us raise awareness and find ways not to get caught in them—either through reformulating goals or changing feedback loops (e.g., adding, altering, strengthening, weakening) [33]. Although system archetypes exist in systems science, we did not find any for CBPR partnerships. Additional study in this area is warranted, which will likely require looking across multiple CBPR partnerships like CHARA.

### Changing or Transcending Paradigms

Meadows identifies changing paradigms and transcending paradigms as the primary leverage points for changing the structure of systems [33]. There may be a paradigm shift underway in research, namely to adjust research priorities in service to learning organizations and, more recently, learning healthcare systems. This presents an opportunity to move beyond some of the siloes in funding and training that create challenges to CBPR: that academics should specialize in an area of disease or a method, that quality improvement and implementation science are distinct, that research and evaluation have different gold standards. Increasingly, funding agencies are encouraging researchers to bridge these gaps through collaboration with patients, communities, or health system partners. We need brave leaders, like CHARA, to establish novel ways of partnering to bridge research and action and to evaluate and publish on these approaches to inform the field.

## Conclusion

In this article, we explored opportunities to utilize concepts from systems science to inform a case study of the development, evolution, and sustainability of one CBPR partnership: the Community Health Advocacy and Research Alliance (CHARA). Systems science provides important tools that can help CBPR partnerships proactively evolve over time. In parallel, CBPR can inform best practices for stakeholder engagement in systems science and traditional scientific inquiry, to bridge the gap between research and practice. Alignment and application of these two complementary approaches can help (1) improve academic-community partnership functioning and sustainability, (2) ensure that research addresses the priorities and needs of end users, and (3) support more timely application of scientific discoveries into routine practice.

Over the past 4 years, CHARA has leveraged initial seed funding from the Patient Centered Outcomes Research Institute (PCORI) Pipeline to Proposal Award Program to develop a robust academic-community partnership that is closely aligned with regional health system transformation in the Columbia River Gorge. CHARA has utilized a CBPR approach to understand regional priorities, build academic and community-based capacity, and pursue research and action. CHARA, and network partners, have secured multiple research and programmatic awards that signify “success.” Yet, like many CBPR partnerships, the CHARA core team and advisory board are still exploring ways to stay financially viable beyond individual projects. This article demonstrates how application of tools and concepts from systems science may support the sustainability of CBPR partnerships by identifying opportunities to strengthen reinforcing and balancing loops, understand adaptive cycles and system archetypes, and support efforts to change or transcend paradigms. We posit that linking CBPR and systems science provides a structure to help academic-community partnerships like CHARA keep their core identities stable even as they co-evolve through interactions with dynamically changing individual, organizational, and national health and social landscapes.

## Footnotes

a
Two community-based members of the CHARA core team (Dillon and Lowe) were participants in the first year of the PACE program. Ms Lowe currently serves as the patient chair for PACE and 2 additional CHARA board members participated in the most recent international PACE training in November 2017.

## Previous Presentations

Elements described in this manuscript were presented at the 2017 Annual Practice-Based Research Network Conference in Bethesda, MD (June 2017) and at the 45th North American Primary Care Research Group (NAPCRG) Annual Meeting in Montreal, Quebec, Canada (November 2017).

## Supplementary material

For supplementary material accompanying this paper visit https://doi.org/10.1017/cts.2018.334. click here to view supplementary material
