# Supplementary material for: Aligning systems science and community-based participatory research: A case example of the Community Health Advocacy and Research Alliance (CHARA)
Source: J Clin Transl Sci. 2019 Feb 5;2(5):280–8. doi: 10.1017/cts.2018.334 (PMC6390389; doi:10.1017/cts.2018.334)
Supplement: Supplementary file 1 [file S2059866118003345sup.zip › S2059866118003345sup001.docx]

**Appendix 1. Nine Principles of Community-based Participatory Research (CBPR)**

| 1. Recognize the community as a unit of identity; 2. Build on community strengths and resources; 3. Facilitate collaborative, equitable partnership in all phases of the research; 4. Promote co-learning and capacity building among all partners; 5. Integrate and achieve a balance between research and action for the mutual benefit of all partners; 6. Emphasize local relevance of public health problems and ecological perspectives that recognize and attend to the multiple determinants of health and disease; 7. Involve systems development through a cyclical and iterative process; 8. Disseminate findings and knowledge gained to academic and community partners; and 9. Commit to a long-term process and group sustainability. |
| --- |

From Israel BA, Schulz AJ, Parker EA, Becker AB, Allen III AJ, Guzman JR. Critical issues in developing and following community based participatory research principles. In: Wallerstein MMN, ed. *Community Based Participatory Research for Health*. San Francisco, CA: John Wiley & Sons, Inc.; 2003:53-76.
