# Supplementary material for: Aligning systems science and community-based participatory research: A case example of the Community Health Advocacy and Research Alliance (CHARA)
Source: J Clin Transl Sci. 2019 Feb 5;2(5):280–8. doi: 10.1017/cts.2018.334 (PMC6390389; doi:10.1017/cts.2018.334)
Supplement: Supplementary file 1 [file S2059866118003345sup.zip › S2059866118003345sup002.docx]

**Appendix 2. The Community Health Advocacy and Research Alliance (CHARA) Regional Foundation.**

The Community Health Advocacy & Research Alliance (CHARA) built on existing relationships and infrastructure in the Columbia Gorge. The Columbia Gorge region spans the Columbia River and includes four counties in north central Oregon and two counties in south central Washington State. These six counties have a combined area of 8,560 square miles and a total population of less than 84,000; only six cities in the region have more than 1,000 residents.

In the Columbia Gorge, and across Oregon, healthcare transformation is underway through implementation of 16 Coordinated Care Organizations (CCOs). CCOs are similar to Accountable Care Organization (ACO), and they are redesigning healthcare delivery and coverage for people eligible for the Oregon Health Plan (Medicaid). CCOs are governed locally by a board of health leaders and community representatives; each CCO is required to engage a community advisory council (CAC) made up of 50% members, operate a clinical advisory panel (CAP) of health system leaders, and conduct a community health needs assessment to inform a community health improvement plan (CHIP) every 5 years. CCO performance is evaluated annual based on performance on 17 quality incentive metrics.

[
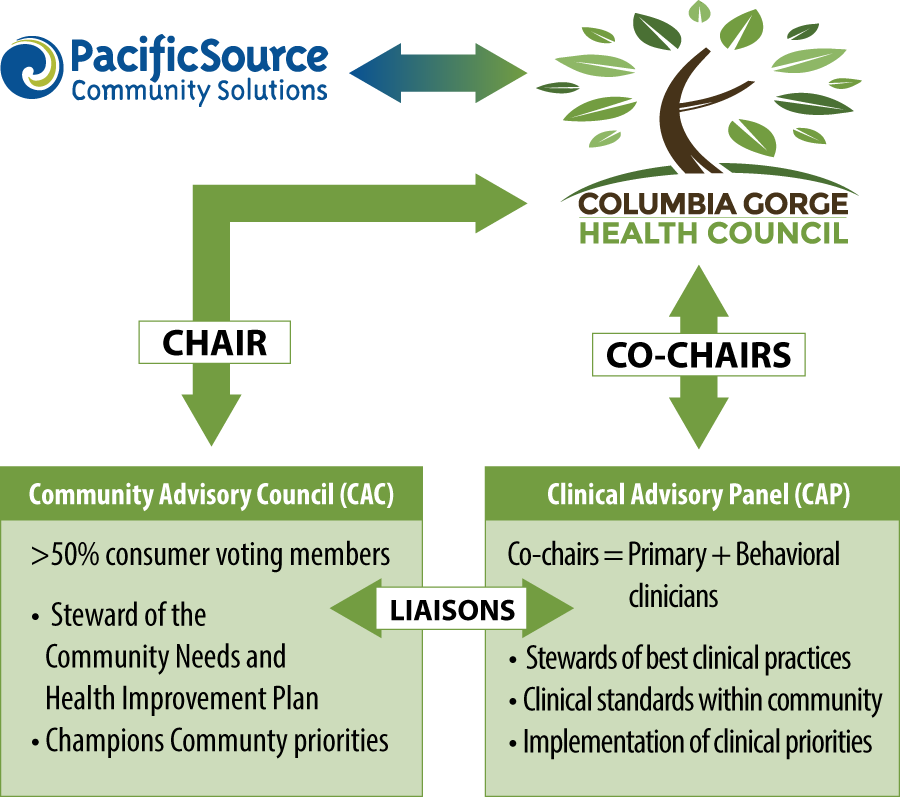
](http://cghealthcouncil.org/wp-content/uploads/2016/09/diagram.png)PacificSource Community Health Solutions Coordinated Care Organization – Columbia Gorge Region was established in November 2012 and administers nearly all Medicaid services for a two-county region of the Gorge (Hood River and Wasco Counties). As summarized in the figure, the Columbia Gorge Health Council works in partnership with PacificSource Community Solutions to operationalize the local CCO. The Columbia Gorge Health Council consists of local leaders in healthcare along with county and community representatives. Thru local engagement and leadership, its mission is to improve health for all of the residents of the region. The Community Advisory Council (CAC) ensures a community voice in programs while the Clinical Advisory Panel (CAP) provides stewardship of best clinical practices. CHARA builds on and complements this existing infrastructure; exploring opportunities to apply research to solve known problems and to conduct research in light of novel questions.
