# Supplementary material for: Aligning systems science and community-based participatory research: A case example of the Community Health Advocacy and Research Alliance (CHARA)
Source: J Clin Transl Sci. 2019 Feb 5;2(5):280–8. doi: 10.1017/cts.2018.334 (PMC6390389; doi:10.1017/cts.2018.334)
Supplement: Supplementary file 1 [file S2059866118003345sup.zip › S2059866118003345sup003.docx]

**Appendix 3. Community Health Advocacy and Research Alliance (CHARA) Mission, Vision, and Values.**

| **Mission** | To identify, develop, and conduct health research to answers questions that matter in the Columbia Gorge region. |
| --- | --- |
| **Vision** | We bring together community members, patients, healthcare professionals, and researchers to collect and generate good research questions in order to learn how to foster health in our community and to share what we learn with others. |
| **Values** | - We define health as a state of well-being for people and as a desired outcome of healthcare and many other social and educational activities - We recognize the health of our community as a regional economic asset. - We harness individual and community strengths as the solutions to local needs. - We practice the principles of collaboration and align our work with others in the community. - We facilitate creation and funding of relevant research questions that test interventions and programs to improve health. - We conduct research that is rigorous, professional, and contributes to the larger body of knowledge about fostering health. |
